# Transition of care from post-acute services for the older adults in Quebec: a pilot impact evaluation

**DOI:** 10.1186/s12913-024-10818-2

**Published:** 2024-04-03

**Authors:** Shashank Ghai, Kathleen Chassé, Marie-Jeanne Renaud, Lilian Guicherd-Callin, André Bussières, Diana Zidarov

**Affiliations:** 1 Department of Political, Historical, Religious and Cultural Studies, Karlstad University, Karlstad, Sweden; 2 Centre for Societal Risk Research, Karlstad University, Karlstad, Sweden; 3 Montréal West Island Integrated University Health and Social Services Centre, Montreal, Québec Canada; 4 School of Physical and Occupational Therapy, McGill University, Montreal, Québec Canada; 5 Centre de Recherche Interdisciplinaire en Réadaptation du Montréal Métropolitain, Montréal, Québec Canada; 6 Departement Chiropratique, Université du Québec à Trois-Rivières, Trois-Rivières, Québec Canada; 7 Faculté de Médicine, Université de Montréal, Montréal, Québec Canada; 8 Centre de Recherche Interdisciplinaire en Réadaptation (CRIR), Institut universitaire sur la réadaptation en déficience physique de Montréal (IURDPM), Centre intégré universitaire de santé et de services sociaux du Centre-Sud-de-l‘Île-de-Montréal, Montréal, Québec Canada

**Keywords:** Post-acute care, Multimorbidity, Geriatric care, Rehabilitation

## Abstract

**Background:**

Early discharge of frail older adults from post-acute care service may result in individuals’ reduced functional ability to carry out activities of daily living, and social, emotional, and psychological distress. To address these shortcomings, the Montreal West Island Integrated University Health and Social Services Centre in Quebec, Canada piloted a post-acute home physiotherapy program (PAHP) to facilitate the transition of older adults from the hospital to their home. This study aimed to evaluate: (1) the implementation fidelity of the PAHP program; (2) its impact on the functional independence, physical and mental health outcomes and quality of life of older adults who underwent this program (3) its potential adverse events, and (4) to identify the physical, psychological, and mental health care needs of older adults following their discharge at home.

**Methods:**

A quasi-experimental uncontrolled design with repeated measures was conducted between April 1st, 2021 and December 31st, 2021. Implementation fidelity was assessed using three process indicators: delay between referral to and receipt of the PAHP program, frequency of PAHP interventions per week and program duration in weeks. A battery of functional outcome measures, including the Functional Independence Measure (FIM) and the Patient-Reported Outcomes Measurement Information System (PROMIS) Global-10 scale, as well as fall incidence, emergency visits, and hospitalizations were used to assess program impact and adverse events. The Patient’s Global Impression of Change (PGICS) was used to determine changes in participants’ perceptions of their level of improvement/deterioration. In addition, the Camberwell Assessment of Need for the Elderly (CANE) questionnaire was administered to determine the met and unmet needs of older adults.

**Results:**

Twenty-four individuals (aged 60.8 to 94 years) participated in the PAHP program. Implementation fidelity was low in regards with delay between referral and receipt of the program, intensity of interventions, and total program duration. Repeated measures ANOVA revealed significant improvement in FIM scores between admission and discharge from the PAHP program and between admission and the 3-month follow-up. Participants also reported meaningful improvements in PGICS scores. However, no significant differences were observed on the physical or mental health T-scores of the PROMIS Global-10 scale, in adverse events related to the PAHP program, or in the overall unmet needs.

**Conclusion:**

Findings from an initial sample undergoing a PAHP program suggest that despite a low implementation fidelity of the program, functional independence outcomes and patients’ global impression of change have improved. Results will help develop a stakeholder-driven action plan to improve this program. A future study with a larger sample size is currently being planned to evaluate the overall impact of this program.

**Clinical trial registration:**

Retrospectively registered NCT05915156 (22/06/2023).

**Supplementary Information:**

The online version contains supplementary material available at 10.1186/s12913-024-10818-2.

## Background

The Canadian healthcare system is facing tremendous challenges as a result of an aging population [1]. While approximately 0.5% of Canadians are between the age of 50 to 74 years, and 2.6% are 75 years or older, these individuals account for nearly half of all hospital days (45.6% and 56.1%, respectively) [2]. In addition, people with higher health care utilization tend to be hospitalized for longer periods of time (mean of two months) [2]. Hospitalization and prolonged acute care stays can lead to a functional decline, one of the most common adverse outcomes of hospitalization [3, 4]. Furthermore, patients often experience prolonged non-medical stays where they occupy beds while waiting for an alternative level of care, such as home care, rehabilitation, or other services [5, 6]. Importantly, post-hospitalization functional decline may be sustained up to one year following discharge, and failure to return to baseline functional status has been associated with increased risk of institutionalization [7], prolonged disability [8], and death [9, 10]. Post-acute care services are often used to address this functional decline post-hospitalization. Instead of being discharged from acute care to home or directly to a nursing home, many seniors first transition to post-acute care to receive specialized inpatient and/or outpatient rehabilitation to regain functional autonomy [11].

Older adults with multimorbidity have substantial health and social needs after discharge from post-acute care facilities [12], such as maintaining or regaining the ability to perform activities of daily living [13]. Patients have also emphasized a strong desire to remain at home with appropriate support rather than being institutionalized [12, 14]. Similarly, social isolation and emotional distress are also widely reported upon discharge from post-acute care, highlighting the need for social support interventions [12, 15]. Support is also needed for personal care tasks, which include assistance with activities of daily living (e.g. bathing or showering) and with instrumental activities of daily living (e.g. light housework), assistance with medication management, or use of assistive devices [16]. To better meet these needs, health care professionals and caregivers highlighted the importance of effective coordination of services and supports (e.g., a case coordinator) and clear care pathways [17].

In Quebec’s conventional care model, particularly within the Montreal West Island Integrated University Health and Social Services Center (MWIIUHSSC), the patient trajectory includes admission to an acute care hospital, then depending on the patient’s needs, the person may be discharged home or can be transferred to internal post-acute rehabilitation services or intensive functional rehabilitation (RFI). After discharge from these services (acute or post-acute), patients may be referred to external outpatient rehabilitation services to improve functional ambulation and community integration. These publicly funded outpatient services include adult ambulatory rehabilitation (SARCA), home care (SAD), day-hospital care, and community reintegration programs. Despite the availability of these models of care, access to these rehabilitation services in Canada remains a challenge [18]. For example, at Local Community Service Centers (LCSCs) in the MWIIUHSSC’s territory, individuals often face long waits of up to 119 days to access outpatient physiotherapy services after discharge from post-acute care. Similarly, a study by Deslauriers, Raymond [19] found a mean waiting time of 7.6 months (range from 0 to 77 months) for outpatient physiotherapy services in publicly funded hospitals in the province of Quebec (Canada). This delay in access to external rehabilitation services is exacerbated by a persistent shortage of healthcare professionals and an increasing demand for services for an aging population [20, 21]. With the goal of optimizing post-acute care physical functioning and functional independence among older adults admitted to acute care or their rehabilitation facilities, the MWIIUHSSC piloted the Post-Acute Home Physiotherapy Program (PAHP). This program was designed as a new clinical care pathway in accordance with the *Cadre de référence Montréalais* [22], and adapted from the Institut national d’excellence en santé et services sociaux (INESSS) guidelines to address the rehabilitation needs of clients and their families by providing center-based or home-based outpatient rehabilitation services and early supported discharge to improve access and continuity of rehabilitation care [23]. The PAHP program was also intended to accelerate the turnover of acute care and rehabilitation beds.

The PAHP program was implemented in November 2018 in the four LCSCs in the MWIIUHSSC’s territory. Although promising, the impact of the PAHP program and whether it addresses the healthcare needs of older people remained unknown for three years post-implementation. Therefore, the current study aimed to assess: 1) the implementation fidelity of the program, which refers to the degree to which the program was delivered as intended [24]; the impact of the PAHP program on the functional independence, physical and mental health outcomes and quality of life of older adults who underwent this program 3) its potential adverse events (incidence of falls, rehospitalizations, and medical consultations); and 4) to identify the physical, psychological, and mental health care needs of older adults after their discharge to home.

## Methods

### Design

This quasi-experimental uncontrolled study with three measurement periods was carried out between April 1^st^, 2021 and December 31, 2021. This study was approved by the Research Ethics Committee of the MWI-IUHSSC (IRB number: SMHC-20-21). The study was retrospectively registered on June 22, 2023 on clinicaltrials.gov (registration number: NCT05915156). All patients signed an informed consent form. A Transparent Reporting of Evaluations with Nonrandomized Designs (TREND) checklist and a Template for Intervention Description and Replication (TIDieR) checklist are provided as Additional Files 1 and 2, respectively. The purpose of using the TREND checklist was to ensure transparent reporting of the current study to improve research synthesis and facilitate evidence-based recommendations [25]. Similarly, the TIDieR checklist was used to provide a structured account of the PAHP intervention evaluated in this study [26].

### Participants and recruitment

Potential participants were first approached by a member of their inpatient rehabilitation team to seek their interest in participating in the study, before being contacted by one of the project leaders (KC, MJR). Patients were eligible if they were discharged home with or without home-care services and could understand French or English. Patients were excluded if they had an unstable medical, psychiatric, and postoperative condition or a severe cognitive impairment (subjective assessment of the rehabilitation team).

### PAHP program (intervention)

The PAHP program was designed to provide physical rehabilitation services to patients recently discharged from acute or post-acute care. In this study, the term “discharged from acute or post-acute care “, specifically refers to individuals who received in-patient rehabilitation services. As part of the PAHP program planning, each patient was to be treated by one of the four physiotherapy technologists assigned to the PAHP program working at the four LCSCs in the MWIIUHSSC’s territory within 48 h of discharge from an acute or post-acute care facility, and to receive home physiotherapy at least three days a week for up to six weeks. Each session lasted between 45 and 60 min, and the same physiotherapy technologist provided care to the patients in their caseload from the admission to the discharge of the patient from the program. The profession of a physiotherapy technologist is specific to the province of Quebec [27]. These professionals become involved in the patient’s care after the patient’s initial assessment by a physiotherapist or a physician. Their responsibilities include analyzing information from the patient’s medical record and using this data to develop, implement, and monitor individualized treatment plans. Physiotherapy interventions provided through the PAHP were tailored by the physiotherapy technologists based to the reasons for the referral and the patient’s needs. For instance, if a patient was unable to climb or descend stairs independently after discharge from acute or post-acute care, the PAHP therapist focused on building lower extremity strength and providing home stair training. In addition, when patients were referred to the PAHP program, the referring therapist sent goals to work on, and the physiotherapy technologist continued home rehabilitation to achieve those goals. The physiotherapy technologist was also independent in evaluating and determining the specific exercises/treatments needed to achieve these goals. In addition, because of their caseloads or other organizational issues (e.g., staff shortages, other responsibilities, etc.) the physiotherapy technologists were not necessarily able to provide the 3 weekly sessions required by the PAHP to all the older adults and had to extend the duration of the program beyond the required 8 weeks in order to meet the patient’s needs and achieve the established functional goals.

### Measuring instruments

Sociodemographics of all study participants enrolled in the PAHP were collected from the participant’s medical record and interview, including age, gender, education, marital status, principal comorbidity, admission diagnosis to post-acute care, type of living arrangement, presence of caregiver/spouse at home, and length of time in the PAHP program. The primary outcome measures in this study are consistent with the goals of the PAHP program and the recommendations of the International Consortium for Health Outcomes Measurement (ICHOM) for core outcome measures to be used in older adults [28]. The two physiotherapists who performed the outcome assessments were different from the physiotherapy technologists who administered the PAHP program.

#### Implementation fidelity

Implementation fidelity of the intervention was assessed using three process indicators:1) delay between referral and the receipt of the PAHP program (24 to 48 h was targeted); 2) frequency of PAHP visits per week (a minimum of 3 interventions/week was targeted) and 3) duration of the PAHP program in weeks (4 to 6 weeks was targeted).

#### Impact of PAHP program

1. The Functional Independence Measure (FIM) assessed the individual’s functional ability. The FIM measures independent performance in the areas of self-care, sphincter control, transfers, locomotion, communication, and social cognition. The FIM instrument consists of 18 items, and each item score ranges from 1 to 7 (a score of 7 is categorized as “complete independence”, while a score of 1 represents “total assistance”). The total score ranges from 18 (the lowest) to 126 (higher scores on the FIM indicate patients with a greater level of independence and less need for assistance). Thirteen of the 18 FIM items are from the motor subscale, and the remaining five items are from the cognitive subscale [29]. It has been shown that the total FIM scores can be treated as interval scores [30].
2. The Patient-Reported Outcomes Measurement Information System (PROMIS) Global-10 (10-item) survey assesses the self-reported perceptions of health, physical, mental and social health, and the quality of life using a 5-point Likert scale. The scale is reliable, precise and comparable to legacy instruments [31]. The PROMIS-10 physical and mental health scores are converted into standardized T-scores [32]. A T-score of 50 is identified as the mean score for the general population, with a higher or a lower score indicating better or worse physical and mental health, respectively.
3. Patient Global Impression of Change Scale (PGICS) (1 item) assessed participants’ perceptions of their level of improvement or deterioration in their physical function at discharge from the PAHP program. An 11-point Likert scale was used with options ranging from “very much worse” to “completely recovered”. The minimum clinically important change is 1.35 points on this 11-point scale [33]. The scale has been previously validated in the context of chronic pain rehabilitation [34, 35].

#### Potential adverse events of PAHP program

Adverse events related to the PAHP program were assessed using fall events at two time points: first, from the time participants were discharged from acute or post-acute care until the end of the PAHP program, and second, at the 3-month follow-up after discharge from the PAHP program. To further evaluate the adverse events associated with the program, patient self-reports of the number of emergency visits and hospital readmissions during these time periods were also analyzed.

#### Needs assessment

The Camberwell Assessment for the Need of the Elderly (CANE) was used to assess the met and unmet physical, psychological, social and environmental care needs (24 domains) from the perspective of the older adults [36]. The CANE has previously been used and validated with older adults living in the community [37–41], and with individuals discharged from acute psychiatric care [42]. For each domain, there is a question about a specific need. Responses are scored on a three-point scale, with 0 indicating no need, 1 indicating met need (problem received appropriate intervention), 2 indicating unmet need (problem left without optimal intervention, and 9 indicating unknown if the patient did not know the type of assistance received.

### Data collection procedure

**Fig. 1:**
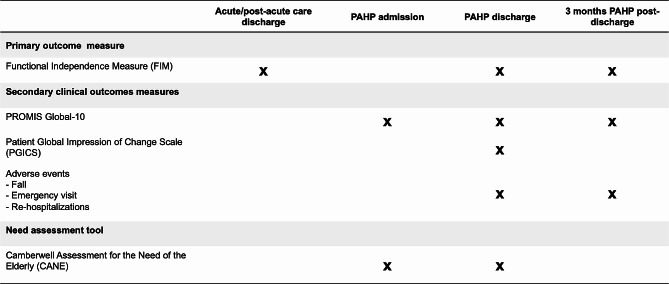
Time periods of data collections

Figure 1 shows the data collection procedure for all the outcomes. The two project leaders, physical therapists by training, collected all outcome measures through face-to-face or telephone interview with participants. They were all trained to administer the FIM and the CANE using the CANE manual [43].

### Data analysis

Descriptive statistics were calculated for participants’ demographic and clinical characteristics, and questionnaire scores using means and standard deviations (continuous variables) and frequencies and percentages (categorical variables). Descriptive statistics were also used to compare implementation fidelity and outcomes related to adverse events (i.e., falls and medical visits related to the primary diagnosis) between two time points: events occurring from acute/post-acute care discharge to PAHP discharge and after PAHP discharge at the 3-month follow-up. We evaluated the impact of the PAHP program on FIM scores and PROMIS-10 (i.e., separately on T-score physical, T-score mental, general health, social activities and role) over time and whether the changes persisted during the 3-month follow-up, using a one-way repeated measures ANOVA with the three time points (i.e., hospital discharge, PAHP discharge, three-month follow-up) as the within-subject factor. When significant effects were observed, post hoc comparisons with Bonferroni correction were performed to determine whether group differences emerged over time. Independent samples t-tests were conducted to evaluate the influence of the PAHP program on needs assessment, including the met and unmet needs as assessed by the CANE questionnaire, across two time points (i.e., PAHP admission and PAHP discharge). The statistical significance level was set at 5%, and all analyses were performed using IBM SPSS Version 28.0 (Armonk, NY: IBM Corp).

## Results

### Demographic and clinical variables

**Fig. 2:**
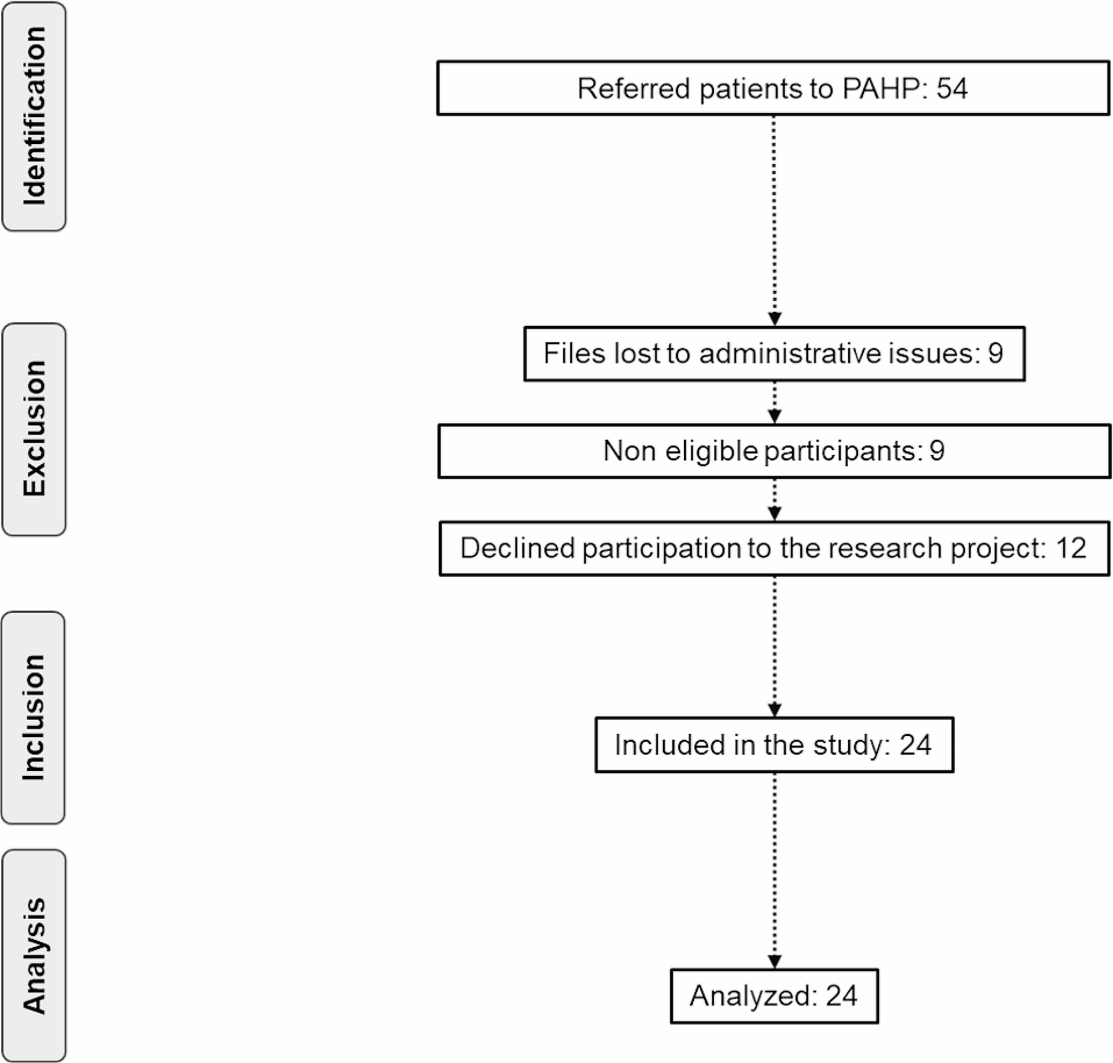
Schematic representation of the included participants

During the study period, 54 patients were referred to the PAHP program of whom 12 declined to participate, nine were ineligible due to cognitive impairment or language barriers, and nine were lost due to administrative issues, leaving 24 (44.4%) study participants (Fig. 2). Data for the three evaluation times were available for 24 participants (50% female, mean age was 80.6 ± 7.9 years). The primary reason for the admission to acute or post-acute care for the patients was musculoskeletal disorders (66.7%), followed by infectious diseases (12.5%), cardiopulmonary disorders (12.5%), neurological disorders (4.2%), and other (4.2%) (Table 1).

**Table 1:** Demographic and clinical characteristics of participants

Characteristics	Mean (standard deviation), or N (%)
**Total participants**	24
**Gender**	
Female	12 (50%)
Male	12 (50%)
**Age (years)**	80.6 ± 7.9 (range: 60.8–94)
**Marital status**	
Single	11 (45.8%)
Married	13 (54.1%)
**Admission diagnosis**	
Musculoskeletal disorder	16 (66.7%)
Infectious disease	3 (12.5%)
Cardiopulmonary disorder	3 (12.5%)
Neurological disorder	1 (4.2%)
Other	1 (4.2%)
**Principal comorbidity**	
Cardiopulmonary disorder	13 (54.2%)
Musculoskeletal disorder	5 (20.8%)
Infectious disease	1 (4.2%)
Neurologic disorder	1 (4.2%)
Other	4 (16.7%)
**Homecare services after discharge**	
With services (home care)	12 (50%)
Without services	12 (50%)
**Caregiver at home**	
Yes	15 (62.5%)
No	9 (37.5%)
**Length of PAHP program (weeks)**	7.8 ± 4.9 (range: 0.57–21.4 weeks)

### Implementation fidelity

Out of the 664 post-acute care users, 54 older adults (8.1%) were referred to the PAHP program. Among these, 24 individuals (44.4%) actively participated in the intervention. The intervention was delivered a mean of 12.5 ± 10 days (ranging from 1 to 48 days) after referral. The PAHP intervention consisted of an average of 1.6 ± 0.72 sessions per week, lasting for a total of 7.85 ± 5 weeks. Patient medical information was available for all participants prior to the intervention. The physiotherapy technologist planned a total of 10.2 ± 2.6 sessions for the participants, while the actual sessions delivered were 10.8 ± 6.6.

### Functional independence measure

The mean (SD) FIM scores at hospital discharge, PAHP discharge, and follow-up were 105.8 (17.5), 111. 2 (13.6) and 111.3 (15.5), respectively (Table 2). The results indicate a significant effect of time of measurement on the FIM scores (F_1.4,30.8_=7.58, *p* = 0.005). Post-hoc comparison with Bonferroni corrections suggests that FIM had improved between PAHP discharge and hospital discharge (*p* = 0.005) and between the follow-up and hospital discharge (*p* = 0.04).

**Table 2:** Descriptive and statistical analysis of outcome measures

Outcome	Hospital-Discharge	PAHP-Admission	PAHP-Discharge	PAHP-follow up	p-value
**FIM (**± SD**)**	105.8 ± 17.5	-	111.2 ± 13.6	111.3 ± 15.5	**0.005**
**PROMIS-10 Global Health**					
**Physical health**					
T-score (± SE)	-	42.6 ± 5.8	43.3 ± 5.1	44 ± 7.5	0.61
**Mental health**					
T-score (± SE)	-	47.6 ± 9.9	46.9 ± 10.0	48.0 ± 8.7	0.91
General health **(**± SD**)**	-	3.1 ± 0.8	2.8 ± 0.7	3.1 ± 0.8	0.59
Social activities & role **(**± SD**)**	-	3.1 ± 1.1	3.0 ± 1.1	3.0 ± 1.1	0.87
**PGICS (**± SD**)**	-	-	1.95 ± 1.3	-	-
**Adverse events**					
**Falls (N)**	-	-	19	9	
Number of individuals	-	-	11	5	
Emergency visits (N)**	-	-	5	3	
Number of individuals	-	-	5	3	
Hospitalizations (N)**	-	-	2	3	
Number of individuals	-	-	2	3	

PGICS: Patient global impression of change scale, ADL: Activities of daily living, CANE: Camberwell Assessment for Needs in Elderly, PROMIS: Patient Reported Outcome Measurement Information System, p-value: associated with analysis of variance, any significant (*p* < 0.05) results indicate differences in scores across the three evaluation periods, **visits in line with the principal diagnosis

### PROMIS-10 global health

The mean PROMIS-10 Global Health T-scores for physical health at admission, discharge, and follow-up were close to normal 42.6, 43.3, and 44, respectively (Table 2). Similarly, the T-scores for mental health at admission, discharge, and follow-up were also close to normal at 47.6, 46.9, and 48, respectively. There were no significant differences between the admission, discharge, and the follow-up T-scores for mental (F_2,44_=0.09, *p* = 0.9) and physical (F_2,44_=0.48, *p* = 0.61) health. For the two subscales that were not included in the T-scores, no significant differences were observed for either the general health subscale (F_2,44_=0.52, *p* = 0.60) or the social activities and roles subscale (F_2,44_=0.13, *p* = 0.87). 

**Patient Global Impression of Change score**.

The average change in the PGICS for older adults participating in the PAHP program was 1.95 ± 1.3.

**Adverse events**.

The number of falls decreased from 19 at post-acute care discharge to nine at the 3-month follow up (Table 2). Similarly, the number of individuals who experienced falls decreased from 11 at PAHP discharge to five at the 3-month follow-up. There was also a reduction in the number of emergency care visits related to principal diagnosis. Here, the number of individuals who had emergency care visits at PAHP discharge was five, which was reduced to three at the 3-month follow up. However, the number of individuals who had a hospitalization related to the primary diagnosis increased slightly at 3 months after PAHP discharge compared to PAHP discharge (2 to 3 individuals).

### Camberwell assessment for needs in elderly

The mean met and unmet needs at the time of PAHP admission and discharge were similar (Table 3). Independent samples t-tests revealed no significant effect of time of measurement on the overall unmet (t_46_=-0.27, *p* = 0.78) and met (t_46_ = 0.37, *p* = 0.70) needs. Regarding specific needs related to the environment, older adults undergoing PAHP had a decrease in unmet needs in areas such as accommodation (% admission vs. discharge: 8.3% vs. 4.2%), looking after home (12.5% vs. 8.3%), and caring for someone (8.3% vs. 4.2%). In addition, decreases in unmet social needs related to daytime activities (20.8% vs. 16.7%) and company (12.5% vs. 8.3%) and physical needs related to self-care (12.5% vs. 0%) were also reported by older adults in the PAHP program. In contrast, increases in unmet physical needs were observed for mobility (16.7% vs. 20.8%), physical health (4.2% vs. 12.5%), eyesight/hearing (0% vs. 8.3%) and medication management (0% vs. 8.3%). Other areas where unmet needs have increased during the PAHP program were food-related needs (environment, 8.3% vs. 12.5%) and memory (0% vs. 8.3%) and psychological distress (psychological needs, 8.3% vs. 12.5%).

**Table 3:** Distribution of met and unmet needs according to Camberwell Assessment of Need for the Elderly (CANE)

CANE sections	PAHP-Admission				PAHP-Discharge			
	Met need		Unmet need		Met need		Unmet need	
	N	%	N	%	N	%	N	%
**Environmental needs**								
Accommodation	5	**20.8**	2	**8.3**	2	**8.3**	1	**4.2**
Looking after the home	20	**83.3**	3	**12.5**	21	**87.5**	2	**8.3**
Food	15	**62.5**	2	**8.3**	14	**58.3**	3	**12.5**
Caring for someone	3	**12.5**	2	**8.3**	2	**8.3**	1	**4.2**
Money/budgeting	5	**20.8**	0	**0.0**	5	**20.8**	0	**0.0**
Benefits	0	**0.0**	0	**0.0**	0	**0.0**	0	**0.0**
**Physical needs**								
Self-care	10	**41.7**	3	**12.5**	9	**37.5**	0	**0.0**
Eyesight/hearing	5	**20.8**	0	**0.0**	4	**16.7**	2	**8.3**
Mobility/falls	19	**79.2**	4	**16.7**	17	**70.8**	5	**20.8**
Incontinence	4	**16.7**	1	**4.2**	3	**12.5**	1	**4.2**
Physical health	17	**70.8**	1	**4.2**	15	**62.5**	3	**12.5**
Drugs	9	**37.5**	0	**0.0**	6	**25.0**	2	**8.3**
**Psychological needs**								
Psychotic symptoms	1	**4.2**	0	**0.0**	1	**4.2**	0	**0.0**
Memory	5	**20.8**	0	**0.0**	5	**20.8**	2	**8.3**
Psychological distress	0	**0.0**	2	**8.3**	1	**4.2**	3	**12.5**
Safety to self	0	**0.0**	0	**0.0**	0	**0.0**	0	**0.0**
Inadvertent self-harm	0	**0.0**	0	**0.0**	0	**0.0**	0	**0.0**
Behavior	0	**0.0**	0	**0.0**	0	**0.0**	0	**0.0**
Alcohol	2	**8.3**	0	**0.0**	1	**4.2**	0	**0.0**
**Social needs**								
Information	5	**20.8**	4	**16.7**	2	**8.3**	4	**16.7**
Abuse/neglect	0	**0.0**	0	**0.0**	0	**0.0**	0	**0.0**
Company	6	**25.0**	3	**12.5**	4	**16.7**	2	**8.3**
Intimate relationships	0	**0.0**	0	**0.0**	1	**4.2**	0	**0.0**
Daytime activities	6	**25.0**	5	**20.8**	8	**33.3**	4	**16.7**
**Overall Mean (%)**	5.7	**23.8**	1.3	**5.6**	5	**21**	1.4	**6.1**
**Standard deviation**	6.2		1.6		6		1.5	

## Discussion

The MWIIUHSSC piloted the PAHP program which reduced waiting times for home physiotherapy rehabilitation to improve the autonomy of older adults transitioning from acute or post-acute care to home. Individuals in the PAHP program showed a significant improvement in functional independence and gains in functional independence were maintained at the 3-month follow-up. Meaningful gains in the PGICS score were also reported by patients following the PAHP program. We observed no statistical differences in the physical and mental health outcomes with the implementation of the PAHP program, nor in the overall met and unmet needs of older adults undergoing the PAHP program.

### Implementation fidelity

Assessing intervention fidelity is critical for understanding how well an intervention was implemented and whether it followed its intended design [44]. In our study, the implementation of the PAHP program was not executed as planned, with the intended 48-hour visit by a physiotherapy technologist delayed by an average of 12 days. The frequency of physiotherapy technologist visits was also lower than intended, with an average of two visits per week compared to the intended three visits per week. Moreover, while the duration of the PAHP program for each participant was close to the intended eight weeks, the duration of the intervention varied between participants, ranging from half a week to 22 weeks. These deviations from the planned frequency of interventions and duration of the program could be due to the varying rehabilitation needs of the patients, which prompted the physiotherapy technologists to adjust the intensity and duration of the interventions offered according to these needs. For example, some patients may have only needed interventions to increase their independence in outdoor locomotor activities, such as endurance walking outdoors or walking on uneven terrain. Others, more deconditioned upon discharge from acute or post-acute care, may have required longer interventions to increase their functional autonomy.

However, despite the deviations from the intended fidelity, it’s important to note that the PAHP program has been sustained for more than 3 years, indicating recognition of its value within the health system. Perhaps, instead of viewing low fidelity solely as a limitation, it can be viewed as an opportunity to adapt the intervention and processes to better meet the needs and realities of the local context, i.e., the LCSCs in the MWIIUHSSC’s territory. For instance, several strategies can be explored to improve fidelity and enhance the effectiveness of the PAHP program. It is critical to assess and address the factors that contribute to delays in referral and delivery of the program. This may include streamlining referral processes, improving communication among multidisciplinary staff, educating clinicians, improving scheduling efficiency, and monitoring quality of care [45, 46].

Ongoing monitoring and evaluation of fidelity, including regular feedback from clinicians and decision makers, could be used to identify areas for improvement and track the impact of changes implemented. This iterative process of adaptation and refinement could not only enhance fidelity but also contribute to a deeper understanding of the effectiveness of the program and its integration into routine practice [47, 48].

### Functional independence

Limited access to outpatient rehabilitation interventions for patients admitted to the acute care setting negatively impacts length of stay and affects continuity of care [49, 50]. This study found an increase in functional independence using FIM after the PAHP program and at 3-month follow-up, which has been associated with improved quality of life [51], and decreased mortality [52]. Small changes in FIM (i.e., as little as one point) are associated with an 8% improved odds of better quality of life [51]. Therefore, these improvements in functional independence support the clinical relevance of the PAHP program, given the improved performance in motor and cognitive subsets associated with functional independence in older adults with multimorbidity. Other studies have also found an association between changes in the FIM and improved balance and mobility outcomes in older adults [53, 54].

### Global impression of change

We also evaluated older adults’ perceptions of improvement/deterioration in physical function following the PAHP program. We observed a clinically meaningful change in perceived improvement in physical function [55, 56]. Improvements in PGICS have been associated with reductions in pain intensity and improvements in treatment effectiveness, mood, and quality of life [57].

### Met and unmet needs

Assessing met and unmet needs from the patient’s perspective is considered essential to better understand whether additional components related to mobility and functional independence need to be added to the PAHP [58, 59], and to allow for effective troubleshooting to make this pilot program more appropriate to the needs of our specific population. We found no significant difference between the overall unmet needs and met needs before and after the PAHP intervention. Further exploration of our findings provide additional insights into the development of effective post-discharge services. First, with regard to environmental needs, we found that older adults had fewer unmet needs related to accommodation, looking after home, and caring for someone after the PAHP program. We also found reduced unmet needs related to daytime activities, company, and self-care. Based on the improved functional independence, it could be interpreted that independence in performing activities of daily living, including self-care, have improved as a result of PAHP and helped this population to better adapt to their accommodations.

However, we also observed an increase in unmet needs for food-related needs, mobility, physical health, eyesight/hearing, medication management, memory, and psychological distress. To address these unmet needs, a standardized assessment of patient needs at discharge could be incorporated into the PAHP program. This strategy could be implemented in an integrated care model with the PAHP program, which could address the unmet needs by preventing and managing declines in the intrinsic capacity and functional ability rather than treating them [60–62]. This is in line with the World Health Organization’s World Report on Ageing and Health and consistent with emerging evidence on the provision of integrated care for older people with complex health needs [61, 63]. The integrated and coordinated services of the PAHP program may involve the integration of different health care professionals, such as nurses, occupational therapists, general practitioners, and social workers, in a synchronized approach to care. This could facilitate the enhancement of intrinsic capacity and address the needs that extend beyond the expertise of a physiotherapist alone. In this way, through collaborative efforts, health care providers can provide personalized and comprehensive care, prioritizing the needs of patients and moving the health care system toward the goal of integrated care for older adults [64].

### Future implications

The PAHP pilot program has the potential to benefit older adults transitioning from acute or post-acute care to their homes and could be implemented in clinical practice. By integrating the program into discharge planning and rehabilitation interventions, older adults could be supported to maintain their functional independence. Likewise, policymakers could consider implementing similar programs to help alleviate the ever-increasing burden on the healthcare system due to an ageing population. Future research could explore the potential impact of such a program on reducing healthcare costs and improving patient outcomes.

The older adults in our study had a range of unmet needs that could not be addressed by physiotherapy alone, which is the only service provided by the PAHP program. Previous reviews have emphasized the importance and benefits of multidisciplinary rehabilitation teams that include nurses, physicians, physiotherapists, occupational therapists, and dietitians. Such an approach has been shown to improve mobility [65], be cost-effective [66], and reduce the risk of mortality and hospital readmissions [67, 68]. Moreover, the presence of comorbidities in this population highlights the need for coordinated care, which could be addressed through the use of a case manager [69, 70]. A post-discharge case manager can help patients achieve autonomy by helping them navigate the healthcare system and access the resources they need to manage their condition and recover at home. A case manager can act as a liaison between the patient and their healthcare team, connecting them with appropriate specialists and community resources such as home health services or support groups. They can also provide guidance on medication management help coordinate follow-up appointments, increase the use of community services, and help patients understand their care plan and what to expect during their recovery. In the case of our study, for example, social services could be directed to individuals with identified needs related to access to food, while a team of physicians and nurses could be assigned to those with needs related to medication management and sensory impairments. Pharmacists could also play a more prominent role in post-discharge services by reviewing medication regimens or addressing issues that may affect patients’ ability to obtain medications [70]. Occupational therapists could play an important role by conducting assessments of individuals and their home environments, arranging or identifying a process for obtaining and installing equipment or modifying homes to prevent falls, and then instructing the patient on its use [71]. In addition, mental health services can be targeted to those with increasing unmet needs related to psychological distress. Previous studies have shown that post-discharge case management is effective in improving clinical outcomes and reducing hospital utilization and re-admissions by improving access to needed services [70, 72].

In addition more participants need to be recruited to assess the overall impact of the program, and future research could examine the effectiveness of physiotherapist-led and interdisciplinary team case management for older adults after hospital discharge [17]. Although not evaluated in the present study, the use of physiotherapy technologist may have provided an alternative model of care that, in addition to reducing costs, could alleviate challenges such as staffing shortages and increased workload for physiotherapists due to growing service demands [73, 74]. Future studies should evaluate the efficacy and cost-effectiveness of integrating physiotherapy technologists into clinical care models to address staffing shortages and rising demands for physiotherapy services. In addition, qualitative research should explore patient and provider perspectives on this alternative care model.

### Limitations

To our knowledge, this is the first study to evaluate the impact of the PAHP pilot program in Quebec. Nevertheless, our study has several limitations. First, the sample was size small which may have resulted in type I or type II errors [75]. Second, due to the COVID-19 pandemic and the highly stressed healthcare system, we faced significant challenges in recruiting and retaining participants. Thirdly, our study used a single-group design without a comparator arm. This lack of a comparator group poses challenges in attributing observed changes solely to the PAHP intervention, as external factors may contribute to the outcomes. However, the decision to use a single-group design was guided by the pilot nature of the PAHP program, which was implemented on a smaller scale to identify potential issues and refine the program prior to broader implementation. Ultimately, our goal is to use these findings to inform the development of a stakeholder-driven action plan aimed to improve the program. Subsequently, we plan to reassess the effectiveness of the program in a larger- trial, this time with a comparator arm for a more comprehensive evaluation. Fourth, our results indicate a mixed level of fidelity in the implementation of the PAHP program. For example, we observed that delays in the program initiation exceeded the intended period, and the frequency of sessions fell short of the intended minimum frequency. These issues, in addition to unmet participant needs will be used to inform a stakeholder-driven action plan to improve this program.

## Conclusion

Findings from this pilot study evaluating the impact of a new accelerated access to care program in Quebec showed improved functional independence and a positive global impression of change among older adults participating in the PAHP program. Our findings provide valuable insights for the development of a stakeholder-driven action plan to improve the program by promoting a multidisciplinary approach and the inclusion of a post-discharge case manager to coordinate healthcare services. Robust research is needed to evaluate the overall impact of this program on healthcare costs and patient outcomes.

### Electronic supplementary material

Below is the link to the electronic supplementary material.

Supplementary Material 1

Supplementary Material 2

## Data Availability

The datasets used and/or analysed during the current study are available from the corresponding author on reasonable request.

## Abbreviations

PAHP: Post-Acute Home Physiotherapy program
FIM: Functional Independence Measure
PROMIS: Patient-Reported Outcomes Measurement Information System
PGICS: Patient’s Global Impression of Change
CANE: Camberwell Assessment of Need for the Elderly
ANOVA: Analysis of Variance
MWIIUHSSC: Montréal West Island Integrated University Health and Social Services Center
INESSS: Institut national d’excellence en santé et services sociaux
LCSC: Local Community Service Centers
ICHOM: International Consortium for Health Outcomes Measurement
